# Effects of iron concentration and DFB (Desferrioxamine-B) on transcriptional profiles of an ecologically relevant marine bacterium

**DOI:** 10.1371/journal.pone.0295257

**Published:** 2023-12-15

**Authors:** Gary R. LeCleir, Jenna Bassett, Steven W. Wilhelm

**Affiliations:** Department of Microbiology, The University of Tennessee, Knoxville, Tennessee, United States of America; Qassim University, SAUDI ARABIA

## Abstract

Research into marine iron cycles and biogeochemistry has commonly relied on the use of chelators (including siderophores) to manipulate iron bioavailability. To test whether a commonly used chelator, desferrioxamine B (DFB) caused effects beyond changing the iron-status of cells, cultures of the environmentally relevant marine heterotrophic bacterium, *Ruegeria pomeroyii*, were grown in media with different concentrations of iron and/or DFB, resulting in a gradient of iron availability. To determine how cells responded, transcriptomes were generated for cells from the different treatments and analyzed to determine how cells reacted to these to perturbations. Analyses were also performed to look for cellular responses specific to the presence of DFB in the culture medium. As expected, cells experiencing different levels of iron availability had different transcriptomic profiles. While many genes related to iron acquisition were differentially expressed between treatments, there were many other genes that were also differentially expressed between different sample types, including those related to the uptake and metabolism of other metals as well as genes related to metabolism of other types of molecules like amino acids and carbohydrates. We conclude that while DFB certainly altered iron availability to cells, it also appears to have had a general effect on the homeostasis of other metals as well as influenced metabolic processes outside of metal acquisition.

## Introduction

It has been known for decades that the concentration, chemical forms, spatial and temporal distributions of oceanic iron are important factors shaping the ecology of marine phytoplankton and the overall productivity of marine ecosystems [1–3]. Iron concentration and availability have broad consequences, affecting everything from the smallest members of the food web to the global climate [4]. Iron is especially critical to marine microorganisms, as it is a key component in heterotrophic and photosynthetic metabolic processes including amino acid synthesis, DNA biosynthesis, methanogenesis, many electron transfer systems, and nitrogen fixation [5, 6].

Due to the low solubility of iron in solution at the pH of seawater [7], dissolved iron accessible by biology (bioavailable) is found at vanishingly low concentrations. This is especially true in open ocean environments far from continental land masses and other sources of iron [4]. Because of its low concentration in the marine water column, iron is frequently the limiting nutrient for primary productivity across large swaths of the ocean often referred to as high-nutrient, low chlorophyll (HNLC) regions [8]. HNLC regions are typically characterized as having sufficient nitrogen (generally in the form of nitrate) available for microbial growth, but a lack of bioavailable iron, which prevents cell densities from reaching the abundances that could be supported by the amount of nitrogen present [9].

Marine bacteria have evolved multiple ways to acquire iron in environments where the dissolved iron supply is low. These approaches include the use of siderophores [5, 10] and heme-mediated iron uptake systems [11, 12]. Some heterotrophic microbes can access iron that is not dissolved in seawater, but rather part of the particulate pool of nutrients. Cordero et al. [13] concluded that bacteria attached to sinking particulate organic material (POM) can actively use iron solubilized by extracellular siderophores regardless of whether or not the bacteria synthesized the siderophores. Additional support for the idea that some bacteria can access iron in POM can be found in Fontanez et al. [2015], which showed that live sediment traps were significantly enriched in genes for TonB-dependent iron transporters (TBDTs) that are involved in binding extracellular siderophore molecules [14].

Recent studies of heterotrophic bacterial colonization and decomposition of POM in the ocean found that bacterial communities associated with POM settling through the water column were enriched with members of the Roseobacter lineage of alpha-proteobacteria [15, 16]. LeCleir et al [2014] found high abundances (up to 95% of 16S rRNA gene libraries) of Roseobacters in non-lethal sediment traps that had incubated marine particles for 1–3 days, and Giebel et al. [17] found Roseobacters to be strongly associated with POM in the North Sea. The idea that Roseobacters can access iron from the POM pool is also supported by the work of Hogle et al [2017], who showed that members of the Roseobacter group can extract heme and hemoproteins directly from algal cellular debris.

Not only have Roseobacters been identified as having the tools to liberate iron [12, 18, 19] and other nutrients sequestered in sinking POM [20, 21], but they have also been recognized as being highly abundant members of marine microbial communities, comprising up to 20% of all bacterial cells in coastal marine environments and 15% of bacterial cells in the open ocean [22]. Roseobacters might be successful in these environments because of their relatively large genomes [22] that facilitate the synthesis of enzymes to break down complex organic compounds as well as proteins and other molecules that could aid in the uptake of nutrients, including iron [23]. Heterotrophic bacterial activity (including that of Roseobacters) on particles sinking through the water column is essential to marine ecosystem health because degradative processes release nitrogen, phosphorus, and bioactive trace metals such as iron, magnesium, and cobalt, back into the water column. These various elements are, in turn, used by other organisms for their various forms of cellular metabolism [18, 24].

Desferrioxamine-B (DFB) is a microbially-produced siderophore that was first characterized as a metabolite of *Streptomyces pilosus* [25]. It has subsequently been found to be produced by a wide variety of other organisms, including some actinomycetes, γ-proteobacteria, and even some eukaryotic fungi. Because of its ability to strongly bind iron, it was quickly recognized that DFB had potential medicinal uses. It was approved for clinical use in 1968 to combat iron toxicity from both iron poisoning [26] and hemochromatosis [27]. DFB has a high affinity for iron [28], and binds at a ratio of 1:1. While DFB has been used to therapeutically manipulate iron concentrations in humans for more than 50 years, it has more recently been conscripted into use by microbial ecologists looking to alter concentrations of bioavailable iron in bottle incubations. These experiments have provided insight into the effects of iron concentrations on microbial physiology [29–32].

Motivated by the data presented in [13, 15, 16], we wanted to: 1) evaluate the ability of Roseobacters, specifically *Ruegeria pomeroyi* DSS3 (DSS3) and other Roseobacters to produce and secrete siderophore-like molecules (SLMs) that might be of benefit when growing in low-iron conditions; 2) verify that DSS3 had the ability to assimilate iron bound by SLMs and; 3) investigate how exogenous iron-chelating molecules like DFB impact DSS3 in experimental manipulations of bottle-based cultures. DSS3 was originally isolated from coastal Georgia seawater [33] and was selected to be the main focus of this study because it is easily grown in the lab and was previously found in relatively high numbers in association with particles collected *in-situ* and incubated within non-lethal sediment traps [15]. DSS3 sequences have also appeared in transcriptome libraries made from sediment traps designed to investigate microbial communities growing on sinking particles [16]. We hypothesized that DSS3 would be capable of producing iron-binding molecules and that these molecules could enhance growth in lab-based cultures. We also hypothesized that DSS3 would respond to lowered iron concentrations and/or the presence of DFB in its growth medium and that changes in the nutrient status of its growth medium would result in altered nutrient acquisition strategies. These changes would be manifested by variations in expression of specific genes and gene sets, perhaps affiliated with metal acquisition. We opted to use a transcriptomics-based approach to obtain a broader view of what processes within DSS3 are affected by the addition of DFB and subsequent low-iron concentrations of the growth medium. We present this report to improve the interpretation of experiments using DFB as an iron-availability manipulator in experimental systems.

## Materials and methods

### Roseobacter strains and culture conditions

All Roseobacter isolates used in this study were provided by the laboratory of Professor Alison Buchan (University of Tennessee, Knoxville, TN). All isolates were grown axenically and maintained on yeast, tryptone and sea salt (YTSS) agar plates until used for experiments.

### Chrome azurol S medium culture conditions

To test for SLM production by various members of the Roseobacter clade, we grew 12 strains of Roseobacters to mid-log phase in liquid YTSS medium then spot-plated 10 μL of the cultures onto YTSS + chrome azurol S (CAS) agar plates [34]. Prior to pouring the plates, we adjusted the pH of the YTSS-CAS medium to 7.5 using NaOH to support Roseobacter growth. Plates were incubated at 30° C for 48 h. When bound to iron, CAS medium has a blue color, and when iron is removed from the CAS-Fe complex, the medium changes from blue to orange. The presence of orange halos surrounding our colonies was indicative of the production and secretion of high-affinity iron-binding molecules.

### Culture conditions for DSS3 during iron uptake and transcriptomic experiments

*R*. *pomeroyi* DSS3 was initially grown axenically in an artificial seawater medium (ASW medium) [35] supplemented with 10 mM acetate as the sole carbon source. Trace metals, except for iron, were also added to the medium. Because no exogenous iron was added to our culture medium, the only source of iron for cells to grow came from trace iron contamination in other reagents or iron that was released from glass culture vessels [36]. For all our experiments, glassware was acid washed prior to usage and Milli-Q water was used to prepare all reagents [37].

### Production of SLMs by DSS3

Siderophore-like molecules (SLMs) were isolated following protocols we have previously employed for marine microbes [38]. To obtain sufficient quantities of SLMs, 10 L of ASW was inoculated with DSS3. After inoculation, the culture was placed on a shaker, at room temperature, for 48 h. The culture was centrifuged at 3700 x G for 15 minutes to remove cells. The supernatant was subsequently passed through a 47-mm diameter, 0.22-μm pore-size polycarbonate filter to remove any remaining cells or debris. The filtrate was acidified to pH 3.0 to protonate Fe-SLM bonds and free the SLMs. This acidified filtrate was gravity fed through an XAD-16 resin column overnight to bind the SLMs (and other dissolved organic matter) from the filtrate. The column was air dried, and the material bound by the resin was eluted from the column using 100 mL of methanol (MeOH). The MeOH was evaporated off using a rotary evaporator. The dried material was resuspended in 1 mL of MeOH and stored at 4° C until future analysis.

### DSS3 SLMs impact on iron uptake

To determine the impact that DSS3 SLMs had on the ability of DSS3 to acquire iron, we set up four different experimental cultures that consisted of ASW medium with varying levels of iron and DSS3 SLMs using radioactive ^55^Fe as a tracer. DSS3 SLM concentration in our solutions was estimated using a standard curve made with the CAS reagent [34]. The four cultures were: 1) high-iron (1 μM FeCl3) with DSS3 SLMs (100 nM); 2) high-iron (1 μM FeCl_3_) without DSS3 SLMs; 3) low-iron (0 μM FeCl_3_) with DSS3 SLMs (100 nM); and 4) low-iron (0 μM FeCl_3_) without DSS3 SLMs. These four cultures of DSS3 were incubated in the presence of ^55^Fe. After a four hour incubation, DSS3 cells were filtered onto 0.2 μM polycarbonate filters and washed with an oxalate reagent according to [39] and assayed for radioactivity using a Perkin Elmer 2910 TR liquid scintillation counter. Cell abundances were measured for the four different cultures using a Leica DMRXA epifluorescence microscope and SYBR green DNA stain. Radiation on the filters (measured as DPM) was normalized to cell counts and reported as fg/ cell.

### Transcriptomes of DSS3 incubated under different levels of iron stress

**Fig 1:**
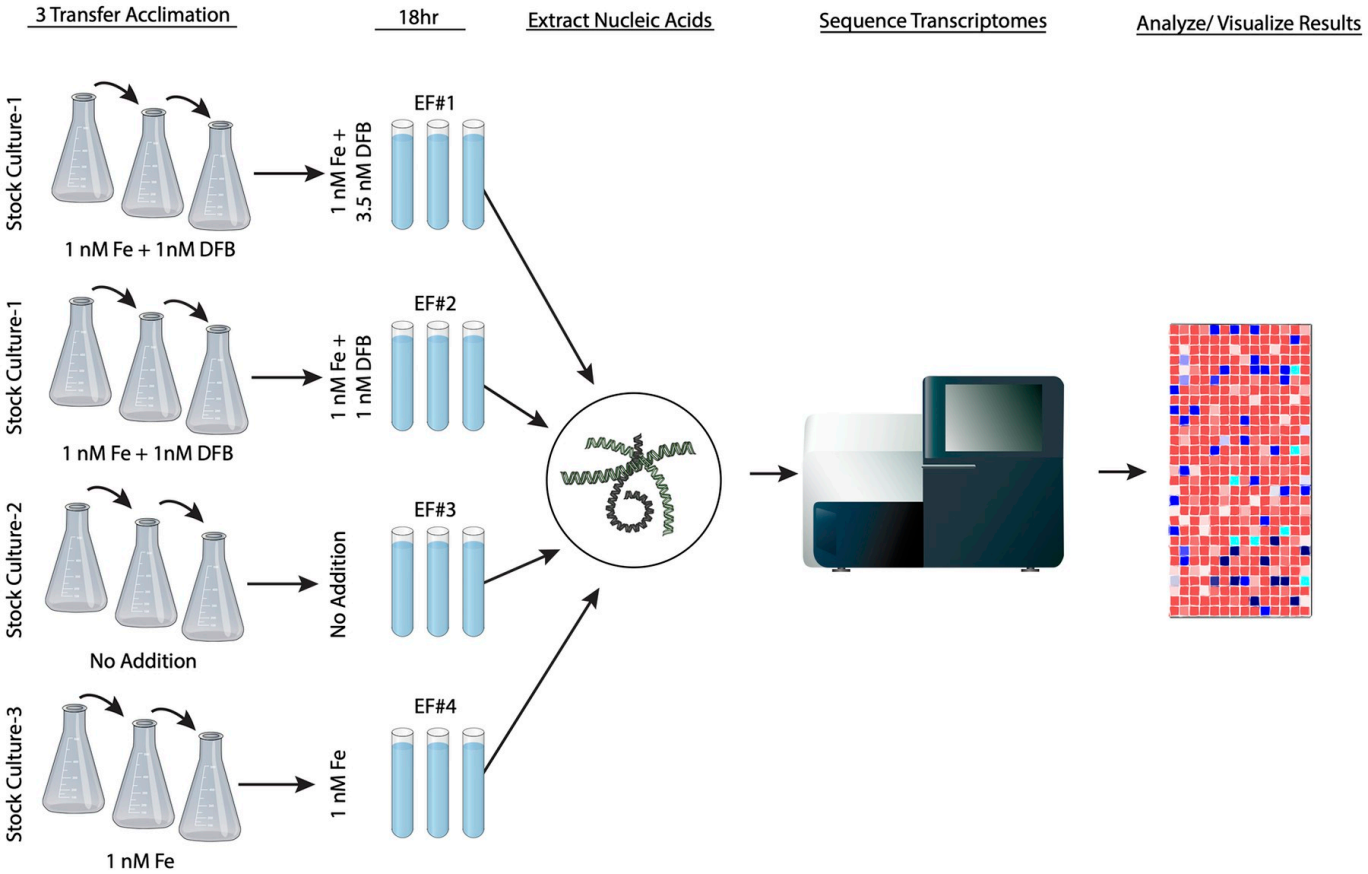
Experimental design and sampling schematic. Three different stock cultures were used to inoculate 4 sets of experimental flasks with varying levels of iron and/ or DFB. After incubation for 18 hours, nucleic acids were extracted, and mRNA was sequenced and analyzed.

DSS3 was initially inoculated into three axenic “stock cultures” (SC) to physiologically equilibrate cells to growth conditions (Fig 1). These cultures were supplemented with different concentrations of iron and/or DFB: SC #1 contained 1 nM Fe, 1 nM DFB; SC #2 contained no added Fe or DFB; SC #3 contained 1 nM Fe and 0 nM DFB.

Stock cultures (SC) were grown overnight and transferred daily into fresh medium of the same composition. This was repeated for three consecutive days prior to the transcriptome experiments. After the three-day acclimation period, aliquots of specific bacterial SC were transferred to triplicate “experimental flasks” (EF) containing the same medium as the SC but with varying amounts of DFB and iron. EF #1 received inocula from SC #1 and contained 1 nM Fe + 3.5 nM DFB to produce a condition of depleted iron relative to the initial SC #1 medium (referred to going forward as the iron-stressed treatment). EF #2 contained 1 nM Fe + 1 nM DFB and received inocula from SC #1 (referred to as low-iron-1); media in EF #3 contained, 0 nM DFB + 0 nM Fe and received inocula from SC #2 (referred to as low-iron-2). EF #4 contained no DFB and 1 nM Fe and received inocula from SC #3 (referred to as iron-replete), (Fig 1). All cultures were grown at 30° C for 18 h. An 18 h incubation time in this medium results in cell cultures in mid-exponential growth phase [40]. After 18 h, cultures were filtered through 0.2 μM pore-size Sterivex^™^ filter cartridges. Filters were frozen at -80° C until further processing.

### Nucleic acid extraction and sequencing

RNA was extracted from the cells collected on Sterivex^™^ filters using a step-by-step, publicly available phenol-chloroform extraction protocol [41]. After nucleic acid extraction was completed, a Turbo DNA-free^™^ kit (Ambion^®^) was used for residual DNA removal. Complete enzymatic degradation of genomic DNA was confirmed via a 16S rRNA gene PCR screen. Removal of genomic DNA was confirmed *via* the absence of an amplicon band in an agarose gel after 30 cycles of PCR amplification using 519F/785R 16S rRNA gene primers as reported previously [42]. If a PCR product was detected, those samples were subjected to a subsequent round of DNAse treatment. All samples were verified to be free from genomic DNA within two rounds of DNAse treatment. The Hudson Alpha Genome Sequencing Center (now Discovery Life Sciences; https://www.hudsonalpha.org/gsc/) performed rRNA reduction and mRNA transcript library sequencing using a 2 x 100 nucleotide protocol on the Illumina Novaseq platform. Nucleotide sequences were deposited in GenBank and assigned the following bioproject accession number: PRJNA954405.

### Bioinformatic analyses

CLC Genomics Workbench package (version 21) was used to filter out poor quality reads and remove rRNA gene related sequences. Reads were then mapped to the *R*. *pomeroyi* DSS3 genome [43]. To account for differences in library sizes, we normalized data as transcripts per million reads (TPM) for each gene. We used the TPM value determined for each gene to calculate Log_2_ fold changes (Log_2_FC) for each transcript across treatments compared against the TPM values for EF #4 (iron-replete) replicates.

### Gene set response to iron stress

TPM values for gene transcripts assigned to 41 gene sets were square root transformed to diminish the impact of extremely high TPM values. The genes that made up these gene sets were previously assigned to these categories according to their gene ontologies [44]. These 41 gene sets were built from an NCBI gene2go data table which was downloaded in September 2013 [45]. The 41 gene sets selected were chosen for analyses because they represented a spectrum of relatedness to iron associated processes. Many of the gene sets were related to processes directly involved with iron acquisition or metabolism (*i*.*e*.,: “metal ion binding” and “metal ion transport”) and other gene sets (*i*.*e*.,: “bacterial flagellum” or “cation sugar symporter activity”) were not related or associated with Fe in any obvious way (see S1 Table for a summary of the gene sets used in this study). Bray-Curtis resemblance matrices were made of the transformed data and these matrices were compared to one another and analyzed to look for differences in expression patterns of these gene sets across treatment types using a pairwise analysis of similarity (ANOSIM) program within the Primer-e (version 7) software package [46].

### Global transcriptome response to iron stress

Primer-e [46] was also used to evaluate transcriptome-wide profile shifts. TPM values for transcripts from all genes of DSS3 were square root transformed and a Bray-Curtis resemblance matrix was made to facilitate the comparison of our different sample libraries using a non-metric multidimensional scaling analysis. Genes with Log2FC -1≥X≥1 and a p-value of less than 0.1 were recorded.

### Identification of iron-related genes and their response to iron stress

To identify genes in the *R*. *pomeroyi* DSS3 genome [43] related to trace element homeostasis, we used the software program FeGenie [47]. Genes identified by FeGenie were analyzed for their responses between treatments in a similar fashion to the comparisons made for the global transcriptome analysis of transcript libraries (see above).

## Results and discussion

Studies by Fontanez et al. [16], Cordero et al. [13] and LeCleir et al. [15] raised the possibility that Roseobacters might be capable of secreting siderophores or SLMs to facilitate iron acquisition under low-iron conditions, thereby providing them a competitive advantage in these types of environments. To investigate the ability of members of the Roseobacter clade to produce SLMs, we plated 12 different Roseobacters, comprising seven different genera (S2 Table), onto Chrome Azurol S (CAS) plates to identify SLM secretion, indicated by the formation of orange halos around the bacterial colonies (S1 Fig). All 12 of our isolates produced and secreted SLMS. The chemical forms and/ or amounts of SLMs produced by our isolates varied as evidenced by different sized halos on the CAS plates. *Ruegeria* sp. TM1040, *Ruegeria pomeroyi* DSS3 and *Loktanella* sp. SE62 produced the largest halos on CAS media. We chose *Ruegeria pomeroyi* DSS3 to continue our investigation into the production of SLMs and the impacts of various iron conditions on the transcriptome of a member of the Roseobacter group because of its prominence in previous studies related to particle colonization and iron acquisition and because it produced a substantial halo on CAS media (S1 Fig).

**Fig 2:**
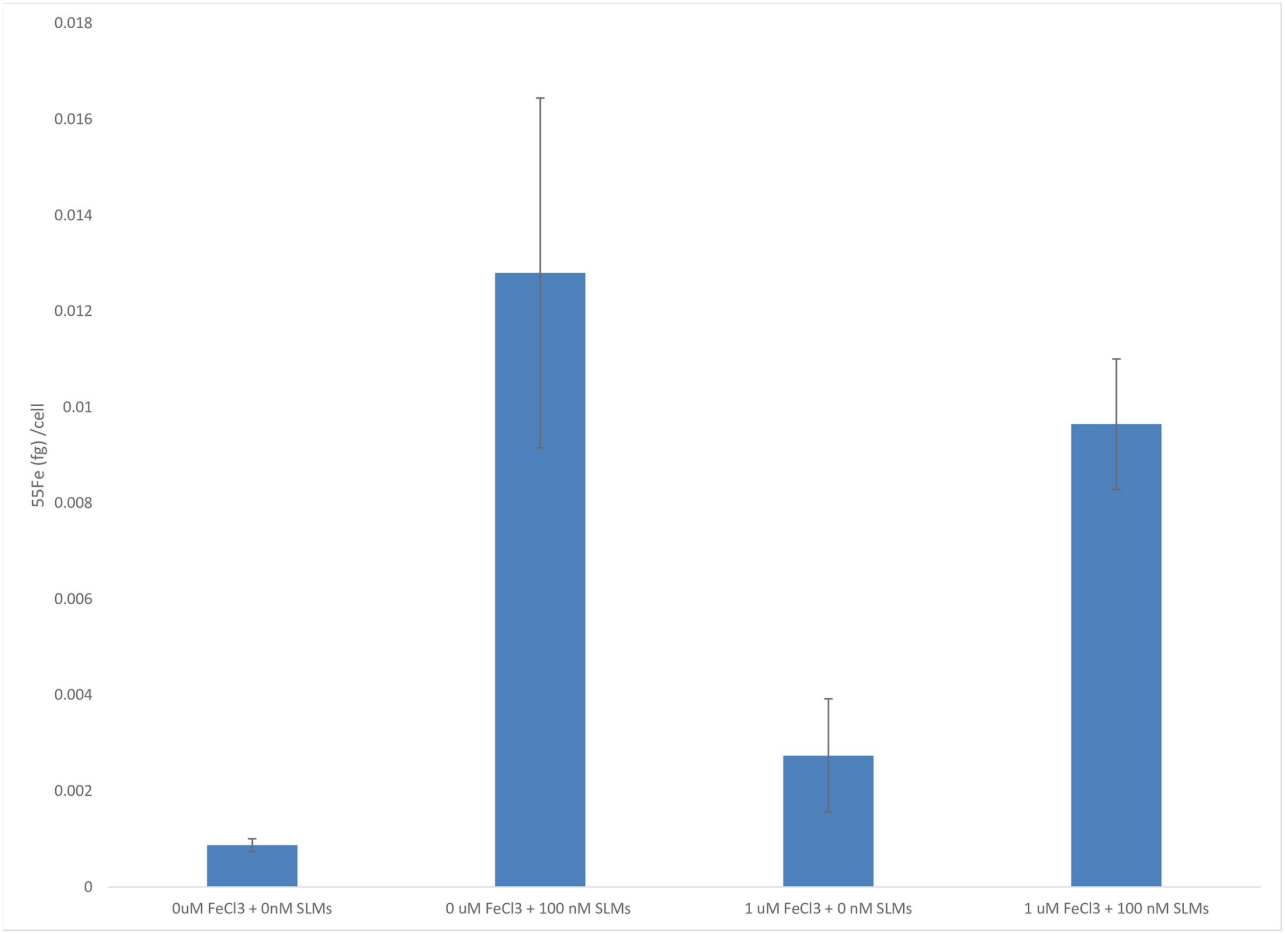
Siderophore-like molecules facilitate enhanced iron uptake. Cultures of DSS3 cells that were grown in either low or high-iron media were able to acquire iron more effectively when supplemented with SLMs than cultures of DSS3 cells that were not supplemented with SLMs.

Culture-based experiments using ^55^Fe and exudates of DSS3 confirmed that DSS3 produces, secretes and can reacquire SLMs when grown under low-iron conditions. When SLMs collected from DSS3 grown under low-iron conditions were added to different DSS3 cultures growing in media with varied iron concentrations, the addition of SLMs resulted in a dramatically enhanced ability of the supplemented DSS3 cultures to acquire iron from the culture medium. DSS3 grown in low-iron medium supplemented with SLMs was able to uptake 14.7 times more iron per cell from the culture medium than the same organism grown in medium without added SLMs (see Fig 2). DSS3 grown in a culture medium with higher iron concentrations was also able to uptake more iron per cell when amended with SLMs; the addition of SLMs to the higher iron medium allowed DSS3 cells to take up 3.5 times more iron per cell than cultures not amended with SLMs (see Fig 2). The ability of DSS3 to produce, secrete and then acquire SLMs to enhance iron uptake may contribute to its capability of growing to high abundances in live sediment traps with large amounts of POM [15].

Although it is genomically well-equipped to grow under a range of environmental conditions, the growth of *R*. *pomeroyi* DSS3 was impaired in iron-stressed experimental cultures (*i*.*e*., DFB 3.5 treatments) when compared to low-iron (*i*.*e*., No Add, DFB 1) and iron-replete (*i*.*e*., Fe) cultures. The average optical density (OD) at 540 nM after 18 hours of growth for iron-stressed cultures (DFB 3.5) was 0.0833. Average OD for the low-iron-1 cultures (DFB 1) was 0.105, for low-iron-2 cultures (No Add) was 0.115 and for the iron-replete treatments (Fe) was 0.116 (see S3 Table). Lower OD is generally indicative of lower cell numbers (or possibly smaller cell size) which would be expected due to the more challenging growth conditions in the iron-stressed samples [48]. The lower ODs provided confidence that the addition of DFB at a final concentration of 3.5 nM stressed the cells in those cultures in such a way that we might expect to see changes in their transcriptomes, especially when compared to our iron-replete cultures.

The effect of iron stress (and DFB) on our cultures can be determined by looking at the patterns of gene expression within our transcriptomic sequencing data libraries. After quality control, filtering, and mapping, we were left with 318,431,592 reads across 13 libraries (an average of 24,494,738 reads per sample, [see S4 Table]). A visual representation of the differences of the transcriptomic profiles for individual experimental culture flasks can be found in S2 Fig. The nMDS plot of the Bray-Curtis similarity matrix illustrates that transcriptomes of samples from similar culture conditions tended to cluster more closely together, indicating that their transcriptomic profiles were more like those collected from the same (or similar) growth conditions than those of cultures with larger differences in iron and DFB. Because the only differences between any of these cultures are the bioavailable iron concentration and the presence or absence of DFB, we can assume that these variables are driving the differences in transcriptomic profiles.

### Gene set analysis

**Fig 3:**
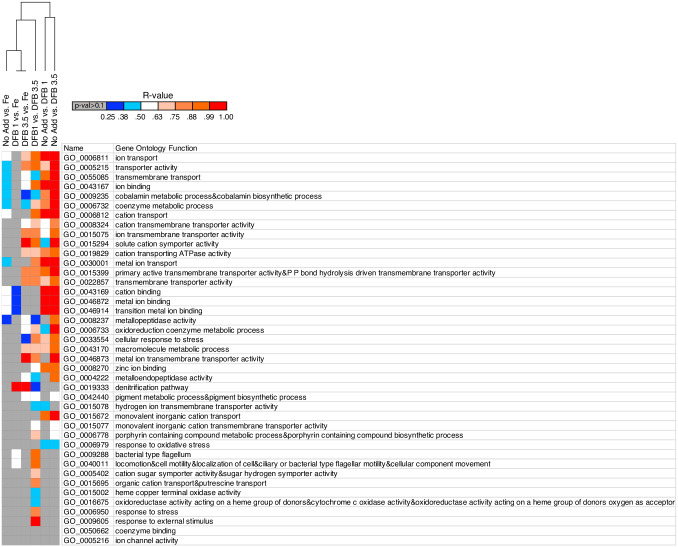
Heatmap displaying the R-values (calculated in primer software package using ANOSIM program) for statistically significant (p-value <0.1) pairwise comparisons between treatments. 125 out of a total of 246 total comparisons were statistically significant at p-value < 0.1.

To determine the effects that iron and DFB concentrations had on cellular functioning (inferred *via* transcript profiles), we examined transcript abundances for gene sets (consisting of genes all assigned to the same gene ontology category (GOs)). In short, genes grouped into the same set and GO category are all related to a common biological process, cellular component, or molecular function [44]. The gene set approach allowed us to investigate how iron stress and low-iron conditions impacted specific cellular processes, components, or functions of DSS3, rather than just the responses of individual genes or groups of unrelated genes. A total of 1,476 unique genes were included in this analysis. Some of these genes were found in more than one gene set, so there was a total of 3,682 data points included in this analysis. These genes were spread across 41 different GOs and were either directly related to cellular metal acquisition, homeostasis and physiology or were related to other cellular functions with varying degrees of relatedness to metals (see Fig 3 and S1 and S5 Tables for gene ontology terms, the number of genes comprising each gene set and the pairwise comparisons and statistical significance of these comparisons).

Fig 3 illustrates that, in general, pairwise comparisons between cultures that were most different with regards to their iron stress differential (ISD) had a higher number of their gene set transcriptional profiles behave statistically different from one another and tended to have the highest number of these statistically significant relationships also have large R-values. R-values approaching a value of 1 indicate increasing differences between samples [46]. ISD was calculated as the potential difference in the perceived iron concentrations experienced by cells of the two cultures being compared. For example, the comparisons between DFB 1 and DFB 3.5 (ISD = 2.5) resulted in RNA transcript profiles for 34 out of 41 gene sets that were statistically different from one another. Whereas comparisons between samples that had similar ISD values (*i*.*e*., DFB 1 to Fe, ISD = -1) resulted in far fewer statistically different gene set profiles (6 out of 41) and few if any R-values greater than 0.75 (see Fig 3). We recognize the relationship between DFB and available iron may not be linear and that ISD is an artificial construct, but this parameter gives us a frame of reference with which to compare samples (see S3 Fig for more information on ISD calculation). Interestingly, the comparison between the two most dissimilar sample types according to ISD (DFB 3.5 and Fe, ISD = 3.5) only resulted in 20 statistically different gene sets and 7 comparisons with R-values greater than 0.75, indicative of a high degree of difference between samples. This was an unexpected result as the differences in Fe availability between these two cultures should have induced the largest alterations in gene expression while the cells adapted to their surroundings to access the elements needed for cellular growth and replication. It is possible that some of the genes involved in responding to varying levels of iron are not included in the gene sets we analyzed. Because there are a large number DSS3 proteins (approximately 40%, http://www.roseobase.org/Species/dss3.html) that are not similar to proteins with known functions, it is possible that some of the genes/proteins involved in Fe metabolism in DSS3 are not yet identified. Another possibility is that DFB does not completely prevent DSS3 from accessing the iron bound to the DFB molecules and so the cells were not as iron-limited in the DFB 3.5 sample as we intended. Although it was previously believed that iron bound by DFB was unavailable for microbial use, more recently it has been suggested that some microbes have access to these chelated iron molecules [49] and can use this iron for growth and physiological processes.

### Differentially expressed genes of the global DSS3 transcriptome

Across the DSS3 transcriptome, there were 157 genes with at least two-fold higher or lower expression in one or more of the low-iron and iron-stressed cultures compared to the Fe-replete cultures (see S6 Table). Of these 157 genes, 134 gene comparisons were supported by p-values < 0.5 and another 23 were supported by p-values <0.1. While 72/ 157 of those genes were over- or under-expressed exclusively in the DFB 3.5 treatment, there were 86/ 157 genes with statistically significant differential expression profiles that occurred in other treatments or combinations of treatments.

**Fig 4:**
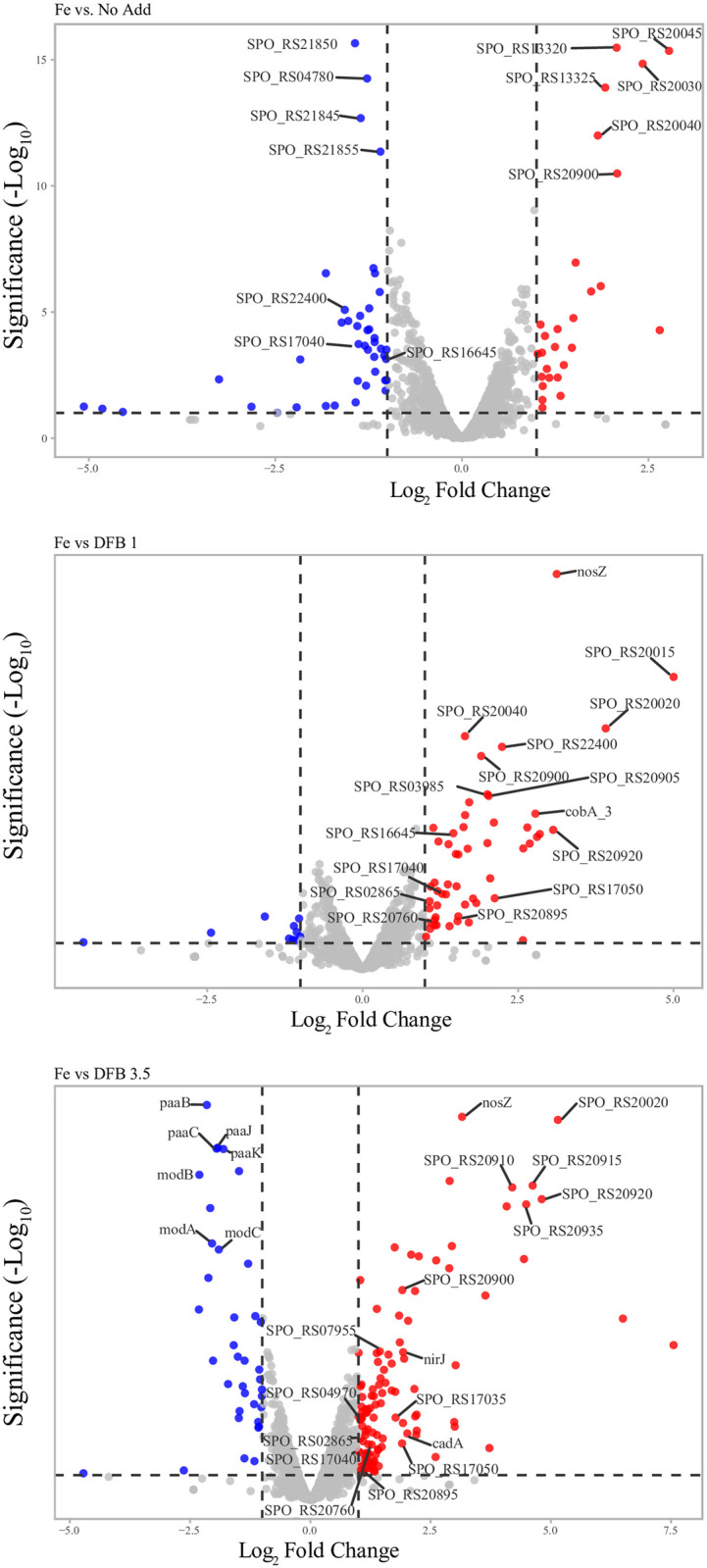
Volcano plots of transcriptomes from iron-stressed cultures compared to Fe-replete cultures. Locus tags were included in the figure for those genes that were either directly related to metal metabolism or ranking based on Manhattan distance (see S7 Table for a list of the genes annotated in the figures).

When assessing the expression of all genes with a significant Log_2_FC, we identified multiple genes related to metal acquisition and iron-related metabolism in our iron stressed and low-iron cultures (for example: SPO_RS16645 (a Fe^3+^ substrate binding protein) and SPO_RS20970 (involved in heme biosynthesis) (See Fig 4 and S6 and S7 Tables). We also noted the presence of many genes related to the acquisition of metals other than iron with significant Log_2_FC expression profiles in our DFB 3.5 treatment (for example: SPO_RS04970 (a zinc ABC transporter substrate binding protein) and SPO_RS17035 (a metal ABC transporter (See Fig 4 and S6 and S7 Tables). Genes encoding zinc transporter binding proteins, cadmium-translocating ATPases, and molybdate transporters and permeases were all differentially expressed in DFB 3.5. Additionally, there were several genes related to cytochrome oxidases, which frequently involve iron molecules as cofactors (see Fig 4 and S6 and S7 Tables) that were differentially expressed. It is not necessarily surprising that genes related to other metals were affected by the presence of DFB because many divalent and trivalent metals can interact with DFB [28] and many proteins can function with multiple metal cofactors during times of elemental stress [50]. These data suggest that the impact of DFB on microbial physiology may be more dramatic and far-reaching than previously appreciated and should be explored in future studies.

Many of the differentially expressed genes from our cultures did not appear to be directly related to Fe or the acquisition or metabolism of other metals, but were involved in other cellular processes (*i*.*e*., carbohydrate (SPO_RS09345 and SPO_RS09350) and amino acid metabolism (SPO_RS05130 and SPO_RS05135) (see Fig 4 and S6 and S7 Tables). These impacts may be due to secondary/ downstream effects of iron stress. For example, when a cell encounters nutrient limitation, the effects of that nutrient limitation may cascade to other processes. If growth and possibly energy production are altered due to metal stress [51–53], demands for additional elements and molecules (*i*.*e*., carbon, nitrogen, amino acids, and carbohydrates) may be altered as well. When demand for these molecules changes, the expression of transcripts encoding the cellular machinery required to obtain and process these molecules may also change [54–56]. Another possibility, as seen in a study of the prymnesiophyte *Phaeocystis antarctica* [57], is that intracellular nutrients and components are diverted away from certain cellular processes and towards others to accommodate for nutrient limitation.

The addition of DFB to culture media, independent of Fe concentration, appeared to have some impact on the transcriptomes of the cells in those cultures, but the response to DFB supplementation was no more dramatic than changes imparted by iron concentration. S2 Fig demonstrates that transcriptomic profiles for all flasks that were amended with some amount of DFB (DFB 1 and DFB 3.5) separate away from the unamended cultures, but all transcriptomes in this study were at least 90% similar to one another. Of note, one of the DFB 1 flasks had a similarity of greater than 95% to two of the Fe amended cultures.

The FeGenie analyses we performed identified 30 genes in the DSS3 genome that were involved in iron acquisition or metabolism. Of these 30 genes, only four were differentially expressed at Log_2_FC values of -1 ≥ X ≥ 1 (S8 Table). An additional six genes were expressed at values -0.58 ≥ X ≥ 0.58 (correlates to fold change expressions of +/- 1.5). We expected a higher percentage of genes identified by FeGenie to be differentially expressed at significant levels, especially because the growth of DSS3 in our iron-stressed culture was so reduced. The analysis suggests that there may be additional genes in the DSS3 genome that are not yet identified as being involved in iron acquisition and metabolism.

The fact that the addition of DFB impaired growth of our DSS3 cultures implies that the iron bound by DFB is less bioavailable to DSS3 than non-bound iron. However, DSS3 retains the ability to import extracellular iron *via* siderophore-like molecules as evidenced by our SLM experiments showing enhanced iron uptake by DSS3 when incubated in the presence of SLMs. We searched the DSS3 genome for evidence of the *desA-desD* genes that are involved in DFB synthesis but found no evidence of those genes in DSS3. We speculate that the SLMs produced by DSS3 are sufficiently different from DFB so that the mechanisms involved in bringing DFB-bound iron into DSS3 do not exist.

In conclusion, DFB and Fe altered gene expression profiles of *R*. *pomeroyi* DSS3. Many of the genes affected by our experimental manipulations were obviously related to metals in some way, but many other genes were also significantly impacted that did not have a direct connection to metal metabolism. Researchers using DFB in their experimental incubations should be cognizant of the downstream effects that iron concentration manipulation can have on bacterial cultures. They should also beware that more than just iron concentration appears to be altered by DFB, as gene transcriptional profiles of many other metal-related transporters and binding proteins were affected by DFB supplementation.

## Supporting information

S1 Fig Roseobacter production of siderophore-like molecules. Twelve different strains of Roseobacters were plated onto chrome-azurol S agar plates. The presence of an orange halo around the colony is indicative of the production and secretion of siderophore-like molecules.
(TIF)

S2 Fig Cultures of DSS3 grown under similar conditions have more similar gene expression profiles. This nMDS plot illustrates that samples from similar culture types tended to cluster more closely to one another. Percent similarity lines encircle samples that are at least that percentage similar to one another.
(TIF)

S3 Fig Calculation of iron stress differential. Relative iron stress was calculated for each pair of cultures to determine the differences in iron concentration experienced between the culture pair.
(PDF)

S1 Table Gene ontology and gene inventories of specific gene sets. (XLSX)

S2 Table Isolates used in this study and their isolation source. (DOCX)

S3 Table Optical density measurements (540 nM) for inoculum and time-final cultures used in these experiments. (DOCX)

S4 Table Sequencing statistics for transcriptomic libraries for different treatments. (DOCX)

S5 Table Statistically significant pairwise comparisons of individual gene sets by treatment. (XLSX)

S6 Table Log2 fold changes and p-values for statistically significant gene expression. (XLSX)

S7 Table Top hits from Fig 4 volcano plots. (XLSX)

S8 Table Log2 fold changes and p-values of FeGenie identified genes compared to Fe treatment. (DOCX)
