# Supplementary figures and images for: Effects of iron concentration and DFB (Desferrioxamine-B) on transcriptional profiles of an ecologically relevant marine bacterium
Source: PLoS One. 2023 Dec 15;18(12):e0295257. doi: 10.1371/journal.pone.0295257 (PMC10723695; doi:10.1371/journal.pone.0295257)

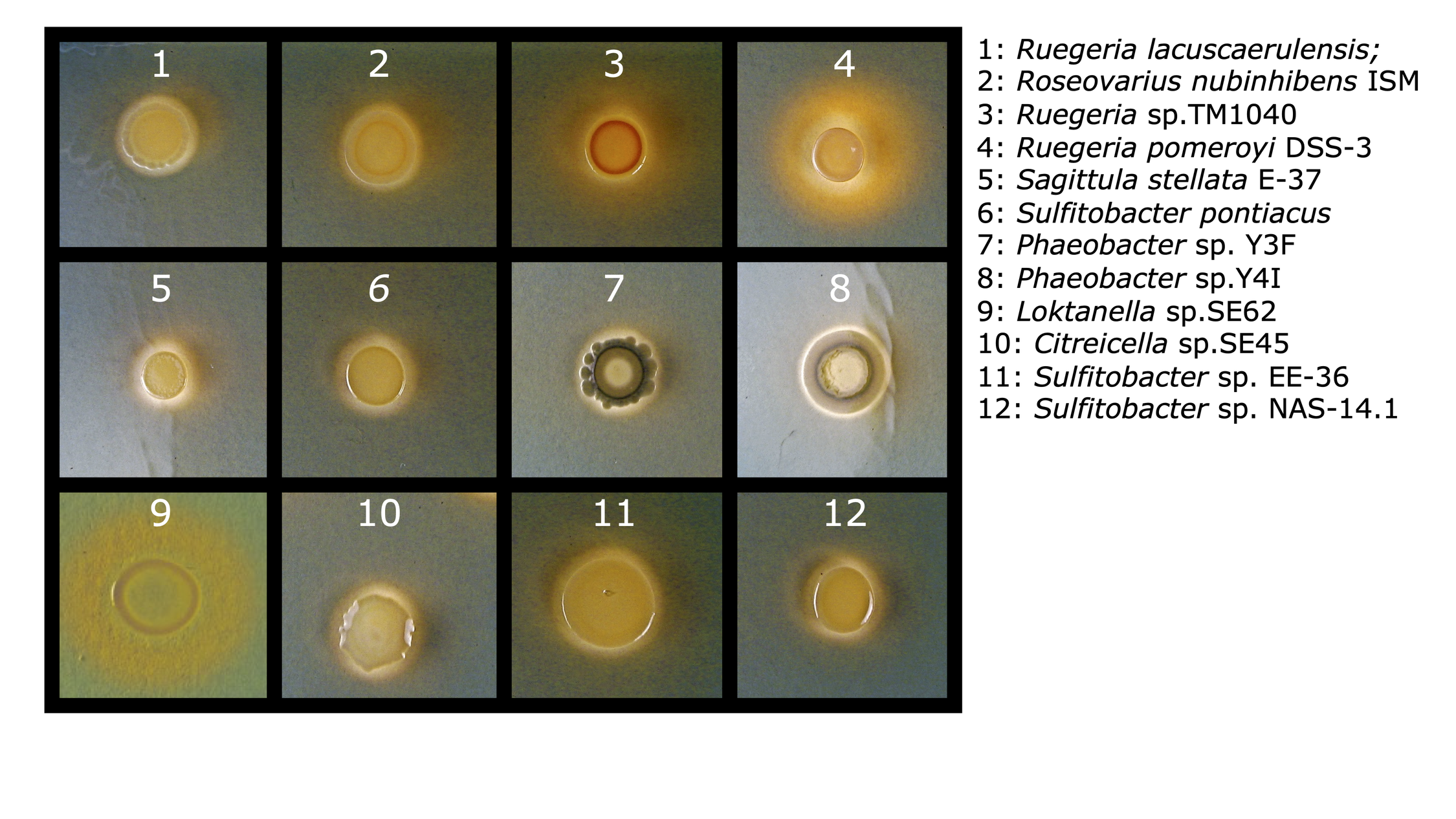

Supplement: S1 Fig — Twelve different strains of Roseobacters were plated onto chrome-azurol S agar plates. The presence of an orange halo around the colony is indicative of the production and secretion of siderophore-like molecules. (TIF) [file pone.0295257.s001.tif]

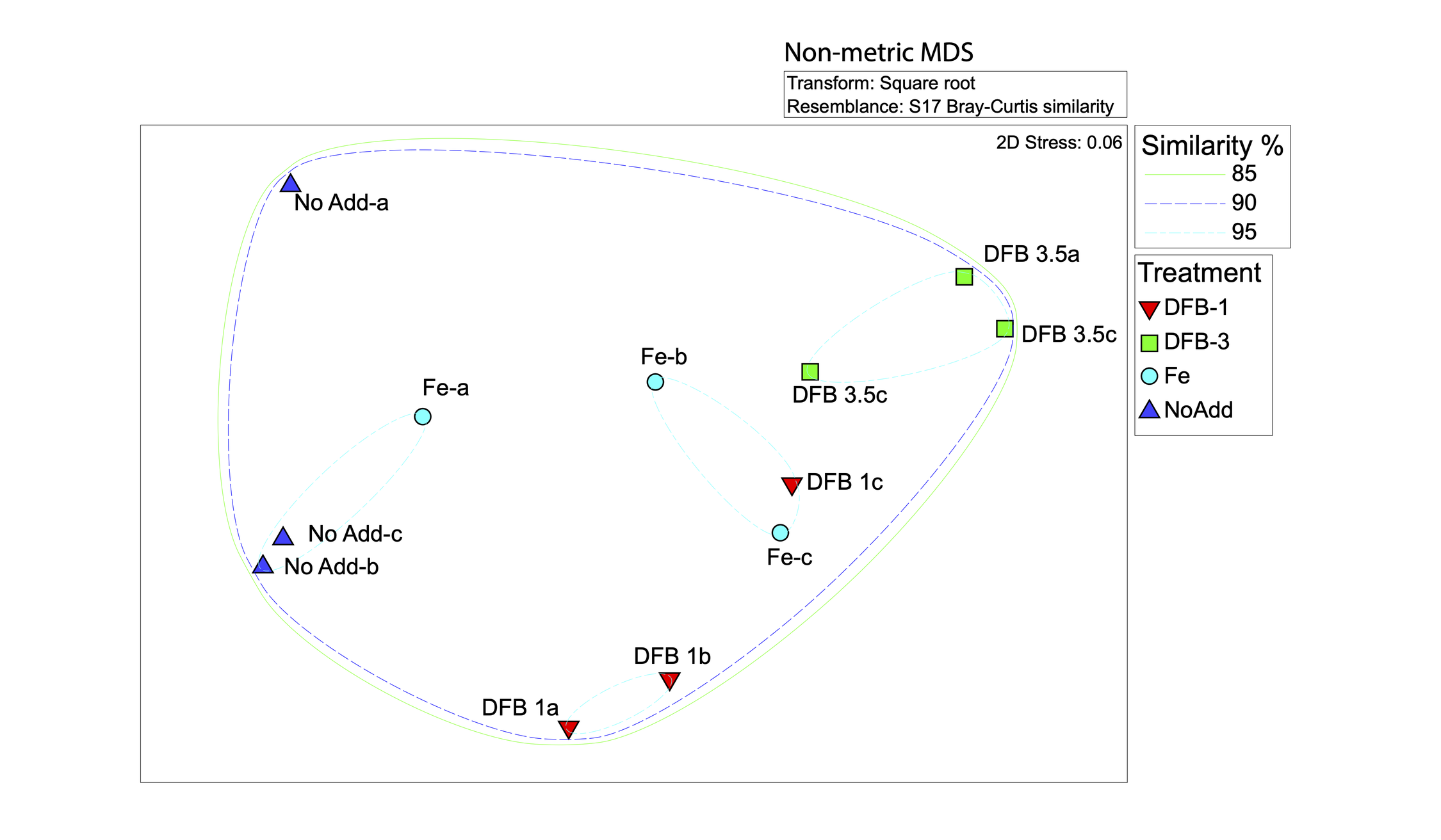

Supplement: S2 Fig — This nMDS plot illustrates that samples from similar culture types tended to cluster more closely to one another. Percent similarity lines encircle samples that are at least that percentage similar to one another. (TIF) [file pone.0295257.s002.tif]
