# Supplementary material for: Effects of iron concentration and DFB (Desferrioxamine-B) on transcriptional profiles of an ecologically relevant marine bacterium
Source: PLoS One. 2023 Dec 15;18(12):e0295257. doi: 10.1371/journal.pone.0295257 (PMC10723695; doi:10.1371/journal.pone.0295257)
Supplement: S3 Fig — Relative iron stress was calculated for each pair of cultures to determine the differences in iron concentration experienced between the culture pair. (PDF) [file pone.0295257.s003.pdf]

Supplementary Figure 3. Calculation of Iron Stress Differential. Relative iron stress was calculated for each pair of cultures determine the differences in iron concentration experienced by the culture pair.

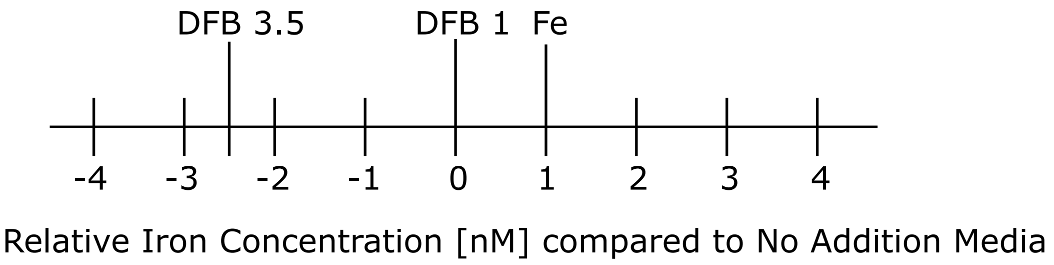

Treatment Pair	Iron Stress Differential (ISD) For Culture Comparisons	ISD
No Add and Fe	0-1	-1
DFB 1 and Fe	0-1	-1
No Add and DFB 1	0-0	0
No Add and DFB 3.5	0-(-2.5)	2.5
DFB 1 and DFB 3.5	0-(-2.5)	2.5
Fe and DFB 3.5	1-(-2.5)	3.5
