# Supplementary material for: Effects of iron concentration and DFB (Desferrioxamine-B) on transcriptional profiles of an ecologically relevant marine bacterium
Source: PLoS One. 2023 Dec 15;18(12):e0295257. doi: 10.1371/journal.pone.0295257 (PMC10723695; doi:10.1371/journal.pone.0295257)
Supplement: S2 Table — (DOCX) [file pone.0295257.s005.docx]

| **Isolate** | **Source** |
| --- | --- |
| *Ruegeria lacuscaerulensis* | Blue Lagoon geothermal lake |
| *Roseovarius nubinhibens* ISM | Caribbean Sea |
| *Ruegeria* sp.TM1040 | *Pfiesteria piscicida* |
| *Ruegeria pomeroyi* DSS3 | coastal Georgia |
| *Sagittula stellata* E-37 | coastal Georgia |
| *Sulfitobacter pontiacus* | Black Sea |
| *Phaeobacter* sp. Y3F | coastal Georgia pulp mill |
| *Phaeobacter* sp. Y4I | coastal Georgia pulp mill |
| *Loktanella* sp. SE62 | decaying *Spartina alterniflora* |
| *Citreicella* sp. SE45 | salt marsh |
| Sulfitobacter sp. EE-36 | salt marsh |
| Sulfitobacter sp. NAS-14.1 | North Atlantic Ocean |

Supplemental Table 2. Isolates used in this study and their source for isolation.
