# Supplementary material for: Effects of iron concentration and DFB (Desferrioxamine-B) on transcriptional profiles of an ecologically relevant marine bacterium
Source: PLoS One. 2023 Dec 15;18(12):e0295257. doi: 10.1371/journal.pone.0295257 (PMC10723695; doi:10.1371/journal.pone.0295257)
Supplement: S3 Table — (DOCX) [file pone.0295257.s006.docx]

Supplemental Table 3. Optical density measurements (540 nM) for Inoculum and Time-final cultures used in these experiments.

| **Treatment** | **Iron Availability Status** | **Inoculum** | **TF (18 hr incubation)** |
| --- | --- | --- | --- |
| Fe-a | iron-replete | 0.089 | 0.114 |
| Fe-b | iron-replete |  | 0.117 |
| Fe-c | iron-replete |  | 0.116 |
|  |  |  |  |
| DFB 1a | low-iron-1 | 0.086 | 0.106 |
| DFB 1b | low-iron-1 |  | 0.103 |
| DFB 1c | low-iron-1 |  | 0.107 |
|  |  |  |  |
| No Add-a | low-iron-2 | 0.086 | 0.11 |
| No Add-b | low-iron-2 |  | 0.112 |
| No Add-c | low-iron-2 |  | 0.122 |
|  |  |  |  |
| DFB-3.5a | iron-stressed | 0.086 | 0.086 |
| DFB-3.5b | iron-stressed |  | 0.081 |
| DFB-3.5c | iron-stressed |  | 0.083 |
