# Supplementary material for: Effects of iron concentration and DFB (Desferrioxamine-B) on transcriptional profiles of an ecologically relevant marine bacterium
Source: PLoS One. 2023 Dec 15;18(12):e0295257. doi: 10.1371/journal.pone.0295257 (PMC10723695; doi:10.1371/journal.pone.0295257)
Supplement: S4 Table — (DOCX) [file pone.0295257.s007.docx]

| **Sample Name** | **Total Reads** | **Quality Controlled and rRNA Depleted** | **Library # from Sequencing Facility** |
| --- | --- | --- | --- |
| DFB 1a | 64,063,354 | 26,390,905 | 448113 |
| DFB 1b | 64,985,534 | 25,291,232 | 448115 |
| DFB 1c | 54,924,124 | 22,028,799 | 448117 |
| DFB 3.5a | 57,812,238 | 25,882,404 | 448123 |
| DFB 3.5b | 65,800,396 | 31,912,614 | 448125 |
| DFB 3.5c | 75,088,586 | 31,373,870 | 448127 |
| Fe-a | 50,686,972 | 20,061,880 | 448128 |
| Fe-b | 63,749,874 | 25,477,487 | 448129 |
| Fe-c | 56,183,058 | 24,403,237 | 448130 |
| No Add-a | 55,266,288 | 20,246,686 | 448131 |
| No Add-b | 60,711,948 | 22,673,691 | 448132 |
| No Add-c | 56,399,510 | 19,630,577 | 448133 |
| No Addition (Technical Replicate) | 58,897,792 | 23,058,210 | 448136 |
| **Total Reads** | **784,569,674** | **318,431,592** |  |
| **Average reads pers sample** | **60,351,513** | **24,494,738** |  |

Supplemental Table 4. Sequencing Statistics for transcriptomic libraries for different treatments
