# Supplementary material for: Effects of iron concentration and DFB (Desferrioxamine-B) on transcriptional profiles of an ecologically relevant marine bacterium
Source: PLoS One. 2023 Dec 15;18(12):e0295257. doi: 10.1371/journal.pone.0295257 (PMC10723695; doi:10.1371/journal.pone.0295257)
Supplement: Supplementary file 7 [file pone.0295257.s011.docx]

Supplemental Table 8. Log2 fold changes and p-values of FeGenie identified genes compared to Fe treatment									
	Log2 Fold Change			p-value < 0.1					
Locus Tag	No Add	DFB 1	DFB 3.5	No Add	DFB 1	DFB 3.5		HMM	Gene Ontology Information
SPO_RS01315				-	-	-		Sid_PchR_pyochelin_regulator	AraC family transcriptional regulator
SPO_RS02875				-	-	-		PF04773_FecR	FecR domain-containing protein
SPO_RS04960			-0.772	-	-	-		FpvD-family-siderophore-transport	metal ABC transporter ATP-binding protein
SPO_RS04965				-	-	-		PF01475-Iron_dependent_repressor-fur_family	transcriptional repressor
SPO_RS04970			-1.013	-	-	0.02		FpvC-family-siderophore-transport	zinc ABC transporter substrate-binding protein
SPO_RS05045				-	-	-		Sid_PchR_pyochelin_regulator	GlxA family transcriptional regulator
SPO_RS07120				-	-	-		Sid_PchR_pyochelin_regulator	helix-turn-helix transcriptional regulator
SPO_RS07140				-	-	-		Sid_PchR_pyochelin_regulator	helix-turn-helix domain-containing protein
SPO_RS07310			0.675	-	-	0.01		Sid_PchR_pyochelin_regulator	GlxA family transcriptional regulator
SPO_RS07600				-	-	-		FpvD-family-siderophore-transport	ATP-binding cassette domain-containing protein
SPO_RS07605				-	-	-		FpvD-family-siderophore-transport	ATP-binding cassette domain-containing protein
SPO_RS07615			-0.58	-	-	0.07		PvdT-family-siderophore-export	ABC transporter ATP-binding protein
SPO_RS08060				-	-	-		Sid_PchR_pyochelin_regulator	helix-turn-helix domain-containing protein
SPO_RS09315				-	-	-		YfeB-family-membrane-proteins	ABC transporter ATP-binding protein
SPO_RS09335	0.864			0.10	-	-		FbpC-family-ATPase	ABC transporter ATP-binding protein
SPO_RS09855				-	-	-		Sid_PchR_pyochelin_regulator	GlxA family transcriptional regulator
SPO_RS12580				-	-	-		PF01475-Iron_dependent_repressor-fur_family	transcriptional repressor
SPO_RS13715				-	-	-		Sid_PchR_pyochelin_regulator	GlxA family transcriptional regulator
SPO_RS14000				-	-	-		Sid_PvdS_regulator_Paeruginosa_PA2426_180620	RNA polymerase sigma factor
SPO_RS14950				-	-	-		Sid_PchR_pyochelin_regulator	AraC family transcriptional regulator
SPO_RS15765				-	-	-		ExbD-family	protein TolR
SPO_RS15770				-	-	-		ExbB-family	protein TolQ
SPO_RS16645	0.875	-1.482	-0.838	0.02	0.00	-		FutA2-family-iron-binding-proteins	Fe(3+) ABC transporter substrate-binding protein
SPO_RS16650		-0.635		-	0.08	-		FbpB-FutB-family-iron-permease	iron ABC transporter permease
SPO_RS16685	-0.854			0.03	-	-		YfeB-family-membrane-proteins	ABC transporter ATP-binding protein
SPO_RS17045		-1.05	-0.667	-	-	-		YfeB-family-membrane-proteins	metal ABC transporter ATP-binding protein
SPO_RS17050		-2.15	-1.871	-	-	0.08		YfeA-family-iron-binding-proteins	zinc ABC transporter substrate-binding protein
SPO_RS17200				-	-	-		Sid_PchR_pyochelin_regulator	helix-turn-helix domain-containing protein
SPO_RS18090				-	-	-		Sid_PchR_pyochelin_regulator	helix-turn-helix domain-containing protein
SPO_RS18315				-	-	-		Sid_PchR_pyochelin_regulator	AraC family transcriptional regulator
